# Protease-Activated Receptor-2 Activation Contributes to House Dust Mite-Induced IgE Responses in Mice

**DOI:** 10.1371/journal.pone.0091206

**Published:** 2014-03-20

**Authors:** Sijranke Post, Irene H. Heijink, Arjen H. Petersen, Harold G. de Bruin, Antoon J. M. van Oosterhout, Martijn C. Nawijn

**Affiliations:** 1 Lab. Allergology & Pulmonary Diseases, Department of Pathology & Medical Biology, University Medical Center Groningen, University of Groningen, Groningen, The Netherlands; 2 GRIAC Research Institute, University Medical Center Groningen, University of Groningen, Groningen, The Netherlands; 3 Department of Pulmonology, University Medical Center Groningen, University of Groningen, Groningen, The Netherlands; National Institute of Environmental Health Sciences, United States of America

## Abstract

Aeroallergens such as house dust mite (HDM), cockroach, and grass or tree pollen are innocuous substances that can induce allergic sensitization upon inhalation. The serine proteases present in these allergens are thought to activate the protease-activated receptor (PAR)-2, on the airway epithelium, thereby potentially inducing allergic sensitization at the expense of inhalation tolerance. We hypothesized that the proteolytic activity of allergens may play an important factor in the allergenicity to house dust mite and is essential to overcome airway tolerance. Here, we aimed to investigate the role of PAR-2 activation in allergic sensitization and HDM-induced allergic airway inflammation. In our study, Par-2 deficient mice were treated with two different HDM extracts containing high and low serine protease activities twice a week for a period of 5 weeks. We determined airway inflammation through quantification of percentages of mononuclear cells, eosinophils and neutrophils in the bronchial alveolar lavage fluid and measured total IgE and HDM-specific IgE and IgG1 levels in serum. Furthermore, Th2 and pro-inflammatory cytokines including IL-5, IL-13, Eotaxin-1, IL-17, KC, Chemokine (C-C motif) ligand 17 (CCL17) and thymic stromal lymphopoietin (TSLP), were measured in lung tissue homogenates. We observed that independent of the serine protease content, HDM was able to induce elevated levels of eosinophils and neutrophils in the airways of both wild-type (WT) and Par-2 deficient mice. Furthermore, we show that induction of pro-inflammatory cytokines by HDM exposure is independent of Par-2 activation. In contrast, serine protease activity of HDM does contribute to enhanced levels of total IgE, but not HDM-specific IgE. We conclude that, while Par-2 activation contributes to the development of IgE responses, it is largely dispensable for the HDM-induced induction of pro-inflammatory cytokines and airway inflammation in an experimental mouse model of HDM-driven allergic airway disease.

## Introduction

Allergic asthma is a chronic inflammatory pulmonary disease that is characterized by airway hyperreactivity (AHR), airway remodeling, eosinophillic and T helper 2 (Th2) cell infiltration into the airways and an allergen-specific IgE response [1]. Inhaled allergens are in first contact with the airway epithelium, which functions as a barrier (towards the inhaled environment) and is an important part of the innate immune system [2]. The airway epithelial response to allergens is considered to be one of the key drivers of airway inflammation in asthma [3]. The aeroallergen House dust mite (HDM) has most commonly been associated with the development of allergic sensitization and asthma [4], [5]. The allergenicity of HDM has largely been attributed to its protease activity, a feature shared by many allergens, including fungi and cockroach [6], [7]. The airway epithelium expresses several so-called pattern recognition receptors (PRRs), which in mouse models were found to be critical for the activation of the airway epithelium by HDM and the induction of an innate immune response [8], [9]. One of the PRRs activated by proteases is protease-activated receptor (PAR)-2, which is expressed by airway epithelium [10] and is up-regulated in the airways asthma patients [11]. PAR-2 is activated by serine proteases present in HDM [12], which stimulate the release of pro-inflammatory cytokines and chemokines including IL-6, IL-8, GM-CSF and TSLP in cultured airway epithelial cells [13], [14].

In mouse studies, inhalation of ovalbumin (OVA) in the presence of a PAR-2 agonist peptide (PAR-2 ap) induced allergic sensitization at the expense of inhalation tolerance [15]. In addition, Par-2 deficient mice showed diminished infiltration of eosinophils and diminished levels of IgE, combined with reduced AHR in the classical OVA-driven experimental asthma model compared to wild-type (Wt) mice [16]. These experiments show that activation of Par-2 may contribute to allergic sensitization through the airways, airway inflammation and AHR upon allergen re-challenge in parenterally sensitized mice. However, no data are available on the relevance for PAR-2 activation in the allergic sensitization driven by HDM, which - unlike the model allergen OVA - harbors endogenous protease activity [17]. Here, we aimed to investigate the role of Par-2 activation in HDM-driven allergic airway inflammation and the induction of an IgE response. To this end, we exposed Par-2 deficient mice to two HDM extracts with low and high serine protease activity [17]. We found that both HDM extracts induced airway inflammation and elevated levels of pro-inflammatory cytokines in lung tissue of Par-2 deficient mice. In addition, exposure to the HDM extract with the high, but not the low level of serine protease activity, increased total but not HDM-specific IgE responses in Par-2 deficient mice. These results indicate that Par-2 activation is largely dispensable for the induction of airway inflammation by HDM and contributes to the induction of an IgE response through activation by serine proteases.

## Materials and Methods

### Experimental Animals

Par-2 deficient mice (B6.Cg-F2rl1tm1Mslb/J) and Wt (C57BL/6J) mice were purchased from Jackson Laboratory (Bar Harbor, Me., USA). Mice were kept under specific pathogen-free conditions and maintained on a 12-hour light-dark cycle, with food and water *ad libitum*. All animal experiments were evaluated and approved of by The Institutional Animal Care and Use Committee of the University of Groningen (The Netherlands).

### HDM sensitization protocol

The biochemical component content in the HDM extracts as well as the administration protocols have been previously described [17]. Briefly, the low serine protease (2.7±1 V/ml, [17]) containing Greer HDM extract (Greer Laboratories, Lenoir, NC, USA) and the high serine protease (2221.9±44.8 V/ml, [17]) containing Citeq HDM extract kindly provided by Citeq Biologics (Citeq Biologics, Groningen, the Netherlands) were dissolved in sterile phosphate-buffered saline (PBS; 2.5 mg total weight/ml) and administered intranasally (20 µl), twice per week for a total of 5 weeks. Twenty-four hours after the last HDM administration, mice were anesthetized with isoflurane/oxygen (Nicholas Piramal India Ltd., London, UK), lungs were lavaged with PBS (UMCG Pharmacy), blood was collected for serum isolation and individual lung lobes were snap frozen in liquid Nitrogen and stored at −80°C until further analysis.

### Collection and measurement of the bronchoalveolar lavage fluid

Briefly, bronchoalveolar lavage (BAL) was performed using PBS, containing 5% bovine serum albumin (BSA) and a mix of protease and phosphatase inhibitors (1 complete mini tablet/10 mL; Roche, Mannheim, Germany). The trachea was cannulated and lungs were lavaged four times with 1 ml PBS. BAL fluid (BALF) cells were pooled and counted using a coulter counter. Cytospin preparations were made and stained with Diff-Quick (Merz & Dade, Dudingen, Switzerland) and evaluated in a blinded fashion. Cells were distinguished into mononuclear cells, neutrophils, and eosinophils by standard morphology. Per cytospin preparation, 300 cells were counted.

### Cytokine assay in mouse lung tissue

Levels of IL-5 and IL-13 in homogenized lung tissue lysates were determined by ELISA, according to the manufacturer's instructions (BD Pharmingen, San Diego, CA). Levels of eotaxin-1, KC, CCL17, TSLP, IFNγ and IL-17 were determined in homogenized lung tissue lysates using Duoset ELISA Development Kit (R&D Systems, Minneapolis, MN), according to the manufacturer's guidelines.

### Measurement of Total- and HDM-specific IgE and HDM-specific IgG1 levels in mouse serum

Total IgE levels and HDM-specific IgE levels were determined as previously described [17]. Briefly, for the total-IgE measurement a NUNC MaxiSorp flat-bottom 96-well plate (Sigma, St Louis, MO) was coated with 1 µg/ml anti-mouse IgE (BD Pharmingen) in PBS overnight at 4°C. Next day, the plates were washed three times with wash buffer (PBS containing 0.05% Tween 20 (Sigma, St Louis, MO, USA)) and blocked for 1 hour with ELISA buffer (50 mM Tris(hydroxymethyl)aminomethane (Merck KGaA, Darmstadt, Germany), 136.9 mM NaCl (Merck KGaA, Darmstadt, Germany), 2 mM Ethylenediaminetetraacetic acid (EDTA; Sigma), 0.5% albumin from bovine serum (BSA; Sigma) and 0.05% Tween, dissolved in 1000 ml H_2_O, pH 7.2) additionally containing 1% BSA. Serum samples and standard (purified mouse IgEκ control) were incubated at room temperature for 2 hours. After the plate was washed three times, samples were labeled with 0.5 µg/ml biotin-anti-mouse IgE (BD Pharmingen,) and incubated for 2 hours. Next, plates were washed three times and incubated with horseradish-peroxidase (1/10000) for 1 hour.

For the HDM-IgE measurement a NUNC MaxiSorp flat-bottom 96-well plate (Sigma, St Louis, MO) was coated with 2 µg/ml anti-mouse IgE (BD Pharmingen) in PBS overnight at 4°C. Next day the plates were washed three times with wash buffer and blocked for 1 hour with PBS containing 1% BSA. Serum samples were incubated at room temperature for 2 hours. After the plate was washed three times, samples were labeled with biotin-conjugated HDM (1/200) and incubated for 2 hours. Next, plates were washed three times and incubated with horseradish-peroxidase (1/300) for 1 hour.

For the HDM-specific IgG1 ELISA, a NUNC MaxiSorp flat-bottom 96-well plate (Sigma, St Louis, MO) was coated with 10 µg/ml HDM (Greer) overnight at 4°C. The plate was washed three times and blocked for 1 hour with ELISA buffer. Serum samples were incubated at room temperature for 2 hours, where after washed three times. Then, samples were labeled for 1 hour with 0.5 µg/ml biotinylated-IgG1 followed by horseradish-peroxidase incubated for 30 minutes.

For all three ELISA's, after the last label step, plates were washed three times and incubated with 0.4 mg/ml *o*-Phenylenediamine dihydrochloride (OPD; Sigma) for about 20 min, where after the reaction was stopped with 4M H_2_SO_4_. The plate was read at 490 nm.

### Immunohistochemistry

Lungs were treated as previously described (17). Briefly, lungs were inflated with TissueTek O.C.T. Compound (Sakura Finetek Europe B.V, Zouterwoude, The Netherlands), and fixed in 10% Formalin for 24-hours, embedded in paraffin and cut in 3 µm-thick sections. Lung sections were deparaffinised in xylene, dehydrated in ethanol and washed in PBS. Antigen retrieval was performed by heating lung sections to the boiling point in 10 mM Tris/1 mM EDTA at pH 9.0 for 15 minutes. Sections were then washed with PBS and blocked with PBS containing 30% H2O2 for 30 min. Lung sections were immunostained with mouse-anti-Par-2 (SAM11; 1/50; Santa Cruz Biotechnology Inc., Heidelberg, Germany) for 1 hour, followed by incubation with the the secondary Ab (1/100; Rabbit-anti-mouse-PO; DAKO, Glostrup, Denmark), and the tertiary Ab (1/100; Goat-anti-rabbit-PO; DAKO). The immunostains were developed by using 3-amino-9-ethylcarbazole (AEC) substrate and mounted with a glass slide using Kaiser's glycerine (Life Technologies Europe BV, Bleiswijk, the Netherlands). Slides were examined and images were acquired by a microscope (Olympus BX53) attached to a Color digital camera (Zeiss) using the Axiovision System (Zeiss).

### Statistical analysis

Statistical significance was determined using the Mann-Whitney-*U* test and P values<0.05 were considered significant.

## Results

### Par-2 deficiency does not affect HDM-induced airway inflammation

**Figure 1:**
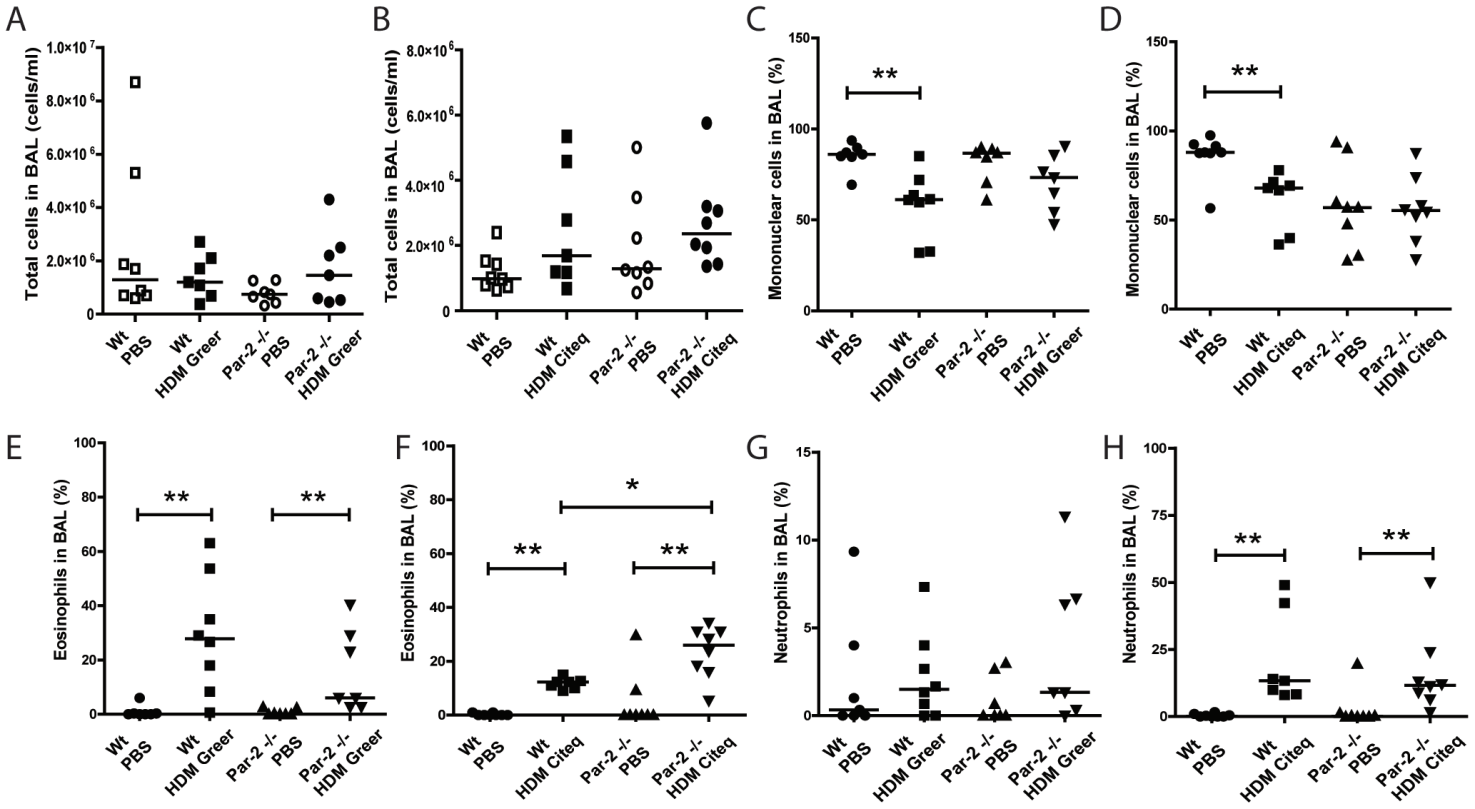
Par-2-deficiency does not influence the inflammatory response after HDM exposure. Total cell counts and mononuclear, eosinophil and neutrophil fractions in BALF from Wt or Par-2 deficient mice after chronic exposure to PBS or HDM (Greer/Citeq). BALF cells were counted 24 hours after the last PBS/HDM exposure. Total cell count after PBS and (A) HDM Greer exposure or (B) HDM Citeq exposure, Mononuclear cell count (%) after PBS and (C) HDM Greer exposure or (D) HDM Citeq exposure, Eosinophil cell count (%) after PBS and (E) HDM Greer exposure or (F) HDM Citeq exposure, Neutrophil cell count (%) after PBS and (G) HDM Greer exposure or (H) HDM Citeq exposure. Median levels are shown (n = 7–8 mice per group). *p<0.05 and **p<0.01 between PBS and HDM exposed mice or differences between Wt and Par-2 deficient mice.

We aimed to test whether activation of the Par-2 by the endogenous serine protease activity of HDM extracts is required for allergic sensitization and the induction of airway inflammation. To this end, we exposed Par-2 deficient or Wt mice twice a week to PBS or HDM extracts with either a low serine protease content (Greer) or a high serine protease content (Citeq) for 5 weeks. We studied inflammatory cells in the BALF 24 hours after the last exposure. Treatment of Par-2 deficient mice and Wt controls with both HDM extracts did not significantly increase total number of inflammatory cells in BALF (Figure 1A and B). Nevertheless, both HDM treatments induced a significant increase in the eosinophilic cell fraction in both Par-2 deficient mice and Wt type controls (Figure 1E and F). Unexpectedly, we observed that the Citeq extract (with high serine protease activity) induced a significantly stronger increase in the percentage of eosinophils in BALF in the Par-2 deficient mice than in the Wt mice (Figure 1F). Only the Citeq HDM extract induced a significant increase in the fraction of neutrophils in BAL fluid, with again no differences between Wt or Par-2 deficient mice (Figure 1G and H).

Furthermore, in Wt mice of the same genetic background as the Par-2 deficient mice (C57Bl/6J) we investigated AHR in response to the Greer HDM extract, which we previously found capable to induce AHR in mice on a BALB/c genetic background [17]. In these experiments, the HDM-treated C57Bl/6J mice did not show significant differences in airway resistance in response to a dilution series of metacholine compared to PBS-exposed control mice (See Figure S1). Given this observation, we did not further analyze AHR in the Par-2 deficient mice. To test whether this difference in HDM-induced AHR between the two strains was dependent on the level of Par-2 expression, we stained lung sections obtained from BALB/c and C57BL/6 mice exposed to PBS and BALB/c mice exposed Citeq and Greer HDM for Par-2 by immunohistochemsitry. We did not observe differences in PAR-2 staining between the bronchial epithelium of PBS exposed C57BL/6 or BALB/c mice (Figure S2A and B), nor did we observe differences in PAR2 staining intensity or pattern between the PBS and Greer or Citeq HDM exposed BALB/c mice (Figure S2A, C and D).

In summary, the HDM-induced eosinophilic airway inflammation was not attenuated, in the Par-2 deficient mice, with even a stronger response in airway eosinophilia induced by the extract with the highest serine protease activity.

### High protease levels are required for allergic sensitization

**Figure 2:**
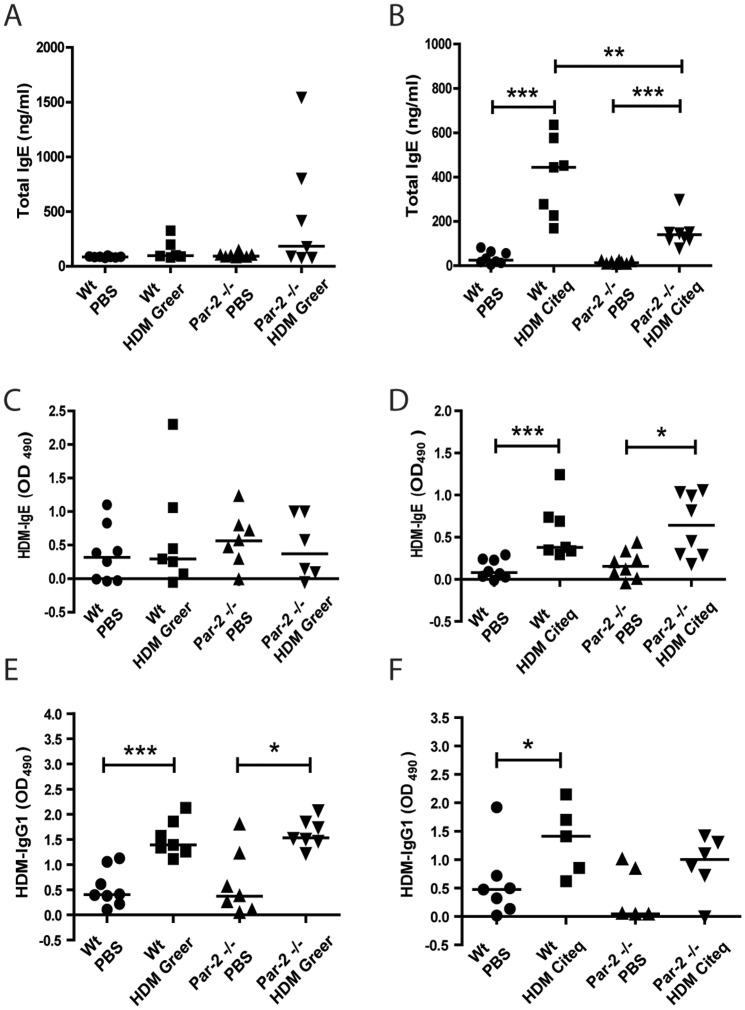
The protease content in HDM is responsible for the IgE but not the IgG1 response. Allergic sensitization response after HDM exposure in Wt and Par-2 deficient mice. Total IgE, HDM-specific IgE and HDM-specific IgG1 ELISA measurements were performed in serum collected 24 hours after the last PBS/HDM exposure. Measurements of total IgE after PBS and (A) HDM Greer exposure or (B) HDM Citeq exposure, HDM-IgE after PBS and (C) HDM Greer exposure or (D) HDM Citeq exposure, HDM-IgG1 after PBS and (E) HDM Greer exposure or (F) HDM Citeq exposure. Median levels are shown (n = 6–8 mice per group). *p<0.05, **p<0.01 and ***p<0.001 between PBS and HDM exposed mice or between Wt and Par-2 deficient mice.

To assess the induction of allergic sensitization after HDM exposure in Wt and Par-2 deficient mice, we first investigated the IgE response. In agreement with our previously reported data obtained in mice with a BALB/c genetic background [17], we observed the induction of an IgE response, both for total IgE levels and for HDM-specific IgE, only in mice exposed to the high serine protease containing Citeq HDM extract, but not in those exposed to the Greer HDM (Figure 2A–D). Interestingly, we observed that these Citeq HDM-induced total IgE levels were significantly lower in Par-2 deficient mice compared to the Wt mice (Figure 2B). Remarkably, this difference was not observed in the HDM-specific IgE response. Next, we investigated the HDM-specific IgG1 response, which showed that both HDM extracts induced significant levels of HDM-specific IgG1 in both Wt and Par-2 deficient mice (Figure 2E–F), although this induction failed to reach statistical significance in Citeq HDM-treated Par-2 deficient mice (Figure 2F).

These results confirm our previous findings in the BALB/c genetic background mice [17], indicating that the high serine protease content in HDM plays a role in the development of the total and HDM-specific IgE response, but not in the HDM-specific IgG1 response. Interestingly, Par-2 deficiency affects the total, but not the HDM-specific IgE response.

### Pro-inflammatory cytokine production in the lung is not affected by PAR-2 deficiency

**Figure 3:**
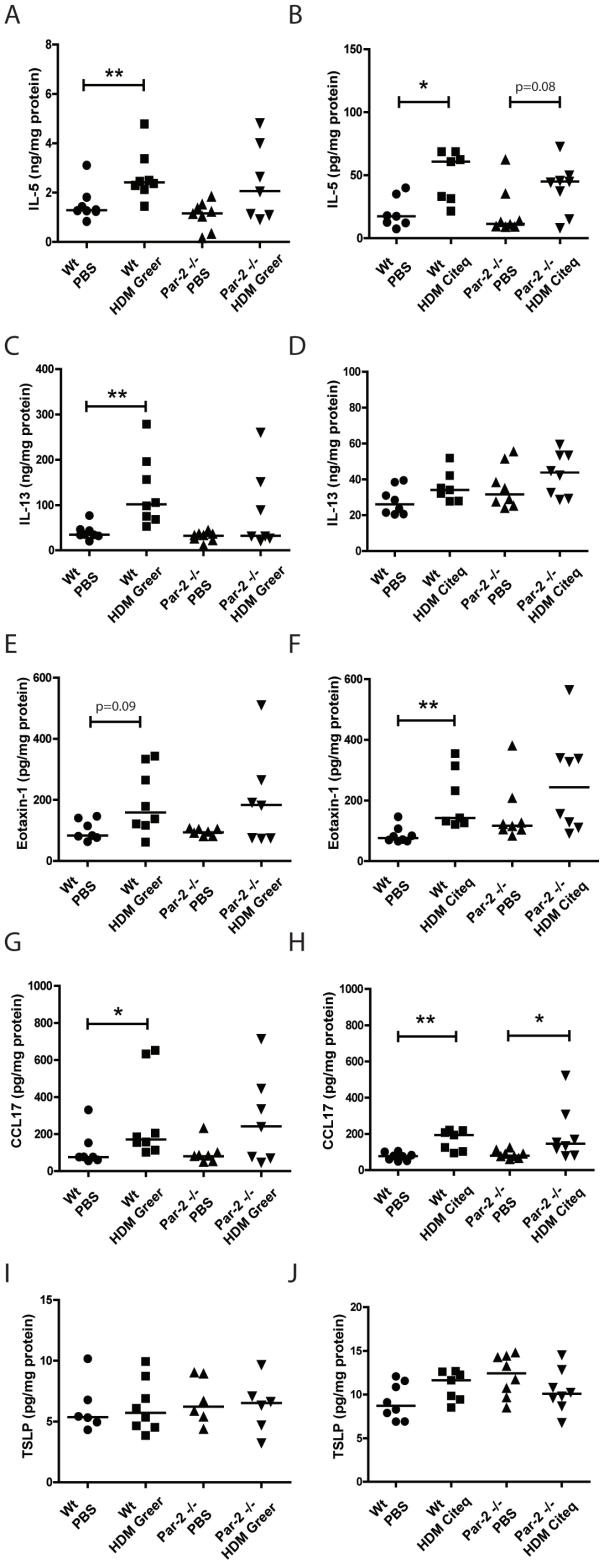
Pro-inflammatory cytokine production is not affected by Par-2 deficiency. Th2 response after HDM exposure in Wt and Par-2 deficient mice. IL-5 and IL-13 ELISA measurements were performed in homogenized lung lysates from lungs collected 24 hours after the last PBS/HDM exposure. Measurement of IL-5 after PBS and (A) HDM Greer exposure or (B) HDM Citeq exposure, IL-13 after PBS and (C) HDM Greer exposure or (D) HDM Citeq exposure, Eotaxin-1 after PBS and (E) HDM Greer exposure or (F) HDM Citeq exposure, CCL17 after PBS and (G) HDM Greer exposure or (H) HDM Citeq exposure, TSLP after PBS and (I) HDM Greer exposure or (J) HDM Citeq exposure. Median levels are shown (n = 6–8 mice per group). *p<0.05 and **p<0.01 between PBS and HDM exposed mice.

Next we assessed the effect of the high and low serine protease extract on levels of Th2 cytokines in the lungs of Wt and Par-2 deficient mice. Both HDM extracts significantly increased levels of IL-5 in the lungs compared to PBS-exposure in Wt mice (Figure 3A–B). In the Par-2 deficient mice, both HDM extracts failed to induce a statistical significant increase in IL-5 levels, although a trend (p = 0.08) was observed for the high serine protease-containing HDM extract (Figure 3B). Furthermore, HDM-induced IL-5 levels were not significantly different between the Wt and Par-2 deficient mice. With respect to IL-13, we only observed a significant increase in Greer HDM-treated Wt mice compared to PBS-exposed mice, with no effect of the Citeq HDM treatment and no differences between Wt or Par-2 deficient mice (Figure 3C–D).

Since IL-5 and eotaxin-1 are both important for the recruitment of eosinophils [18], we also investigated the eotaxin-1 production in both Wt and Par-2 deficient mice after HDM exposure. Only a significant increase in eotaxin-1 levels of was observed in Wt mice exposed to Citeq HDM, while a trend (p = 0.09) towards significance was observed in mice exposed to the Greer HDM extract (Figure 3E–F). Neither extracts were able to induce significant increase of Eotaxin-1 levels in the Par-2 deficient mice (Figure 3E–F), although levels were not significantly lower in the Par-2 deficient compared to the Wt mice.

Next we investigated the expression of the pro-allergic factors CCL17 and TSLP, which attract Th2 cells and promote Th2 cell differentiation, respectively [19], [20]. Here, we found that both HDM extracts induced an increase in CCL17 levels in both Wt and Par-2 deficient mice compared to PBS-exposed mice, with no significant differences between Wt and Par-2 deficient, although this failed to reach statistical significance for the Greer HDM treatment in Par-2 deficient mice (Figure 3G–H). In contrast, the HDM extracts did not induce an increase in TSLP levels in both the Par-2 deficient and Wt groups (Figure 3I–J).

**Figure 4:**
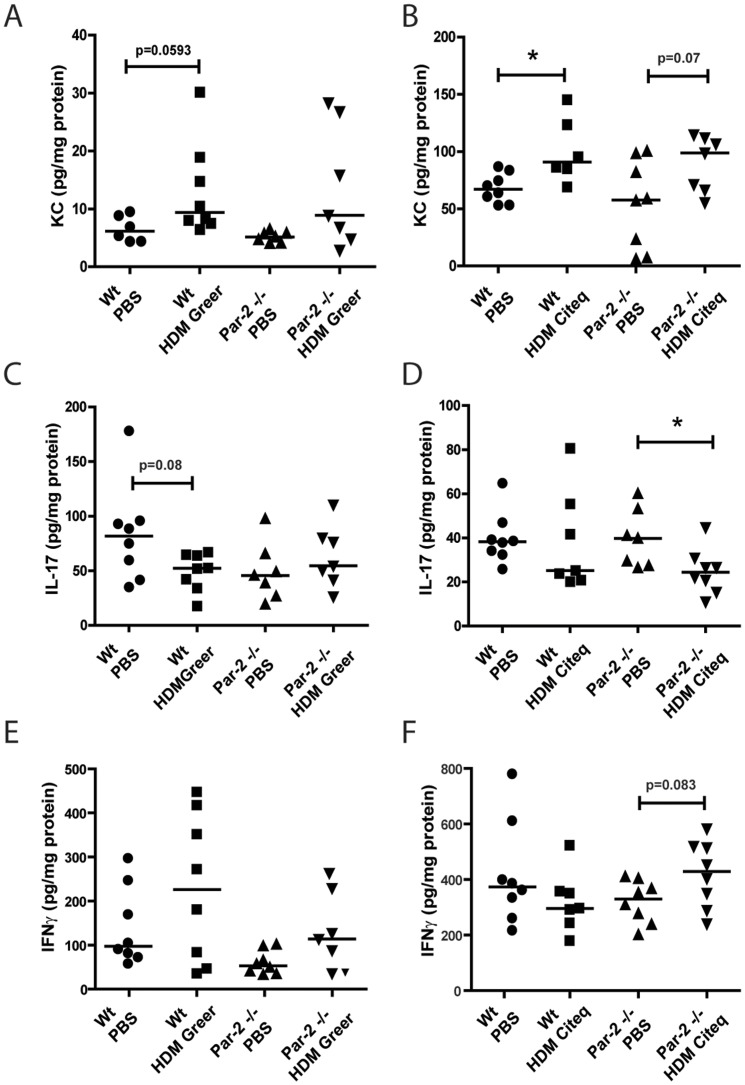
Par-2 deficiency affects IL-17 but not KC or IFNγ production after HDM exposure. ELISA measurements were performed in homogenized lung lysates from lungs collected 24/HDM exposure. Measurements of KC after PBS and (A) HDM Greer exposure or (B) HDM Citeq exposure, IL-17 after PBS and (C) HDM Greer exposure or (D) HDM Citeq exposure, and IFNγ after PBS and (E) HDM Greer exposure or (F) HDM Citeq exposure. Median levels are shown (n = 6–8 mice per group). *p<0.05 and **p<0.01 between PBS and HDM exposed mice.

As we observed induction neutrophillic airway infiltration in Citeq HDM-treated mice (Figure 1H), we also analyzed the levels of KC, a known chemo-attractant for neutrophils [21]. Both HDM extracts induced elevated levels of KC in lung tissue of both Par-2 deficient and Wt control mice compared to the PBS-exposed controls. Although this failed to reach statistical significance in the Par-2 deficient mice (Figure 4A–B), we did not observe significant differences between Par-2 deficient and Wt mice. The differences between the datasets obtained from Citeq and Greer extract exposure may stem from differences in ELISA values due to a different day of analysis.

Subsequently, we investigated levels of IL-17, which is known as a negative regulator for allergic asthma, but positively associated with neutrophillic airway inflammation [22]. Interestingly, HDM treatment generally seemed to down-regulate IL-17 levels, although significance was only reached in Par-2 deficient mice exposed to Citeq HDM (Figure 4D), while a trend (p = 0.08) was observed for the Greer HDM exposure in Wt mice (Figure 4C). Again, we did not observe significant differences between Par-2 deficient and Wt mice.

In comparison, we additionally investigated the Th1 response by analyzing the levels of IFNγ in HDM-treated Par-2 deficient mice and control littermates. In both HDM models, we did not observe an altered level of IFNγ in lung tissue homogenate from wild-type mice, irrespective of the source of the HDM (Figure 4E–F). Also in Par-2 deficient mice, no significant induction of IFNγ is observed for either Greer or Citeq HDM treated mice (Figure 4E–F), although a trend towards increased IFNγ levels is observed in the PAR2-deficient mice treated with Citeq HDM compared to PBS treated controls (Figure 4F).

Overall, these results show that the HDM-induced pro-inflammatory cytokine response in mice is not markedly affected by Par-2 deficiency.

## Discussion

In this study, we investigated the role of Par-2 activation in HDM-induced allergic sensitization and airway inflammation in mice. We show that HDM treatment initiates the influx of eosinophils and neutrophils into the airways, independent of the serine protease levels present in the HDM extract and with no difference between Wt and Par-2 deficient mice [17]. Furthermore, our findings suggest that Par-2 activation may contribute to the IL-13 response in the lungs, although here too, the differences in serine protease activity between the two HDM extracts do not seem to be relevant for the response. In contrast, the level of serine protease activity in HDM does seem to play a role in the development of the total and HDM-specific IgE responses, whereas the presence of Par-2 receptor is only required for optimal induction of total IgE response. Overall, our findings indicate that HDM-induced IgE responses are associated with the protease activity of the HDM extract, while only the total IgE response depends on the activation of the Par-2 receptor. In contrast, the HDM-induced airway inflammation and induction of pro-inflammatory cytokines is independent on Par-2 receptor activation.

The role of PAR-2 in mediating the production of pro-inflammatory cytokines and airway inflammation has extensively been investigated, using both specific small-peptide agonists of the receptor as well as by using Par-2 over-expressing or deficient mice [15], [16]. Schmidlin *et al* were one of the first to show that over-expression of Par-2 in FVB mice leads to exacerbation of airway inflammation in the airways, while deletion of Par-2 in C57BL/6J mice diminishes the inflammation in the airways after OVA exposure [16]. Furthermore, using an OVA tolerance model in BALB/c mice, Ebeling *et al.* showed that administration of an PAR-2 ap simultaneously with OVA administration induced allergic sensitization instead of inhalation tolerance [15], which is induced by OVA when applied through the airways [23]. We addressed the role of Par-2 in mediating airway inflammation using protease-containing HDM, that in contrast to OVA induces allergic sensitization when applied through the airways [17], [24]. Exposure of the Par-2 deficient mice to HDM extracts containing either high or low serine protease activity resulted in airway inflammation, as indicated by the elevated levels of eosinophils and neutrophils in the BALF, with exception for the neutrophil levels after HDM Greer exposure. These results indicate that, unexpectedly, activation of the Par-2 is largely dispensable for the induction of airway inflammation by HDM extracts, at least in the C57Bl/6 genetic background. Thus, other biochemical or molecular components in HDM are likely involved in the development of allergen-induced airway inflammation in mice and/or proteases in HDM are able to provoke airway inflammation without the involvement of Par-2. It is known that HDM extracts contain a wide array of other molecular constituents [17], including the cysteine protease containing allergen *Dermatophagoides pteronyssinus* (Der p) 1 [25], β-glucan structures [9], Der p2 [26] and endotoxin [8] that have all been reported to contribute to the induction of allergic airway inflammation. These compounds act on additional pattern recognition receptors (PRRs) on airway epithelial cells, including the dectin-1 receptor [9] and the Toll-like receptor-4 (TLR4) [8]. We have previously observed that equivalent exposure of LPS in BALB/c mice did not induce production of pro-inflammatory cytokines or airway inflammation compared to HDM exposure [17]. Interestingly, Rallabhandi and co-workers have shown that activation of TLR4 and PAR-2 are cooperative, and that NF-κB activation and subsequent induction of pro-inflammatory cytokine production by TLR4 triggering is first mediated after the engagement of PAR-2 [27]. Although this will need further investigation, possible cooperative TLR-4 and PAR-2 activation, could explain why we observe an increase of pro-inflammatory cytokines in the Par-2 deficient mice after HDM exposure, independent of whether the extract contained high or low serine protease activity, although this failed to reach significance. Furthermore, a recent paper described that another receptor, namely the purinergic receptor subtype P2Y6 (P2Y6R) in C57Bl/6J mice, contributes to increased release of pro-inflammatory cytokines by the airway epithelial cells in response to HDM [28]. Interestingly, we additionally observed down-regulated levels of IL-17 after HDM exposure in both Wt and Par-2 deficient mice, with exception for Par-2-deficient mice exposed to Greer HDM, while we observed increases in levels of CCL17 and Eotaxin-1. Previous studies with IL-17 deficient mice have reported that IL17A is necessary for the development for AHR or airway inflammation in an OVA-induced asthma model, while administration of exogenous IL-17A in OVA-sensitized C57BL/6J mice reduced the levels of RANTES, CCL17 and Eotaxin-1, improving lung function and reduce levels of eosinophils and lymphocytes in the BALF [22], [29]. In addition, Barlow *et al* showed that these protective effects of IL-17A are dependent on the suppression of IL-13 mediated through IL-25 [30]. Since, we did not observe an increase in IL-13 levels in both Wt and Par-2 deficient mice after exposure to both HDM extracts, with exception for Wt mice exposed to HDM Greer, it seems that Par-2 activation may contribute to the IL-13 response in the lungs, independently of the serine protease content in HDM. Furthermore this effect also appears to influence the production of IL-17A negatively, contributing to the allergic pro-inflammatory response to HDM. Consequently, in contrast to the previous *in vitro* studies [13], [14], we conclude that Par-2 activation merely contributes to the induction of pro-inflammatory cytokines *in vivo*, instead of being critically required for the response.

In addition to the role of Par-2 in the HDM-induced airway inflammation, we also studied the role of Par-2 activation in the HDM-induced IgE and IgG1 responses. Here, we found that both specific and non-specific IgE responses were induced by the HDM extract with the high protease activity only, while Par-2 deficiency reduced the total, but not the HDM-specific-IgE response, suggesting that (serine protease-dependent) Par-2 activation is only involved in the HDM-induced total IgE response. In a previous study of Gough *et al*, treatment of Der p1 with an irreversible cysteine protease-specific inhibitor (E-64) reduced the IgE-eliciting activity of the allergen without affecting the production of IgG *in vivo* [24]. In line with these results, we have previously reported that the IgE-inducing high serine protease containing Citeq HDM extract also contains a high amount of cysteine protease activity compared to the Greer extract [17], and we speculate that cysteine proteases may be involved in the HDM-induced IgE responses. In addition, the same study also demonstrated, that mice exposed to an equivalent amount of LPS had no significant effect on IgE levels [17], excluding a role for HDM endotoxins to provoke the observed allergic sensitization response.

For interpretation of our data, it is relevant to consider that cytokine levels in BAL and lung tissue were assessed 24 hours after the last HDM challenge. While we have previously successfully measured HDM-induced cytokine and chemokine responses in lung tissue at this time-point [17], it has been shown that the highest levels after HDM exposure are found 4–8 hours after the last exposure [31]. This indicates that our time-point of analysis might not have been the most optimal for detecting differences in cytokine and chemokine levels in lung tissue or BAL between Par-2 deficient mice and controls. Moreover, we have used C57Bl/6 mice for our analyses. The role of the Par-2 in airway inflammation induced by proteolytically active allergen extracts has previously extensively been investigated in Par-2 deficient mice on both the C57Bl/6J and the BALB/c background, using cockroach fecal remnants (GC frass) as aeroallergen, of which allergenicity is attributed to its serine protease content [32]–[34]. These studies showed that allergic airway inflammation after GC frass exposure is in part mediated via Par-2 in BALB/c but is independent of Par-2 in C57Bl/6J mice [34]. Notwithstanding, also on the BALB/c genetic background, GC frass induced a significant response in Par-2 deficient mice, indicating that GC frass also on the BALB/c background induces an in part Par-2 independent airway inflammation.

The discrepancy between C57Bl/6J and BALB/c in the response to mucosally administered aeroallergen extracts might be due to a difference between the two strains in activation of airway epithelial NF-κB. A recent study by Alcorn *et al* has demonstrated that BALB/c and C57Bl/6J mice differ markedly in their activation of NF-κB in the airway epithelium upon allergen challenge in the classical OVA-driven mouse model of allergic airway inflammation [35]. Here, OVA inhalation in parenterally sensitized mice activates the NF-κB pathway in the airway epithelium of the BALB/c strain but not of the C57Bl/6J strain, which was associated with reduced expression of NF-κB sensitive cytokines induced in the BAL by the OVA challenges in the C57Bl/6J background, most notably TNFα and KC [35]. These data might well be very relevant for the difference in the Par-2 dependence of the response to GC frass between the two strains, since GC frass has been found to induce NF-κB activation [34]. For the data presented here on HDM-induced allergic airway inflammation, it is relevant to note that the role of airway epithelial NF-κB activation for HDM-induced responses was recently found to limited for the classical parameters of allergic airway inflammation: airway epithelial-specific inhibition of NF-κB only partially affected airway eosinophilia, and had no effect on levels of IL-13, HDM-specific IgE and IgG1 and mucus metaplasia after HDM exposure [36]. Therefore, although we have not performed experiments on HDM-induced responses in PAR-2 deficient mice on a BALB/c background that would be more sensitive to airway epithelial NF-κB activation, we propose that our data do clearly indicate that part of the HDM-induced response is independent on Par-2 activation.

Overall, our study shows that Par-2 activation through HDM contributes to the induction of total, but not allergen-specific IgE responses while the production of pro-inflammatory cytokines and the development of airway inflammation is at least in part independent on the activation of Par-2.

## Supporting Information

Figure S1 **C57Bl/6J mice do not show significant differences in airway resistance after PBS or HDM exposure.** Airway hyperreactivity was measured by Flexivent in response to metacholine in Wt C57Bl/6J mice after PBS and HDM Greer exposure. Absolute mean values (±SEM) are shown.
(TIF)

Figure S2 **HDM exposure has no effect on Par-2 expression.** PAR-2 staining of histological lung sections obtained from (A) BALB/c and (B) C57BL/6J mice exposed to PBS and BALB/c mice exposed to (C) Greer HDM extract and (D) Citeq HDM extract twice a week for a period of 5 weeks. Representative pictures are shown. Magnification 40×. *Red arrows* indicate highly PAR-2 expressing cells.
(TIF)
