# Supplementary figures and images for: Protease-Activated Receptor-2 Activation Contributes to House Dust Mite-Induced IgE Responses in Mice
Source: PLoS One. 2014 Mar 20;9(3):e91206. doi: 10.1371/journal.pone.0091206 (PMC3961228; doi:10.1371/journal.pone.0091206)

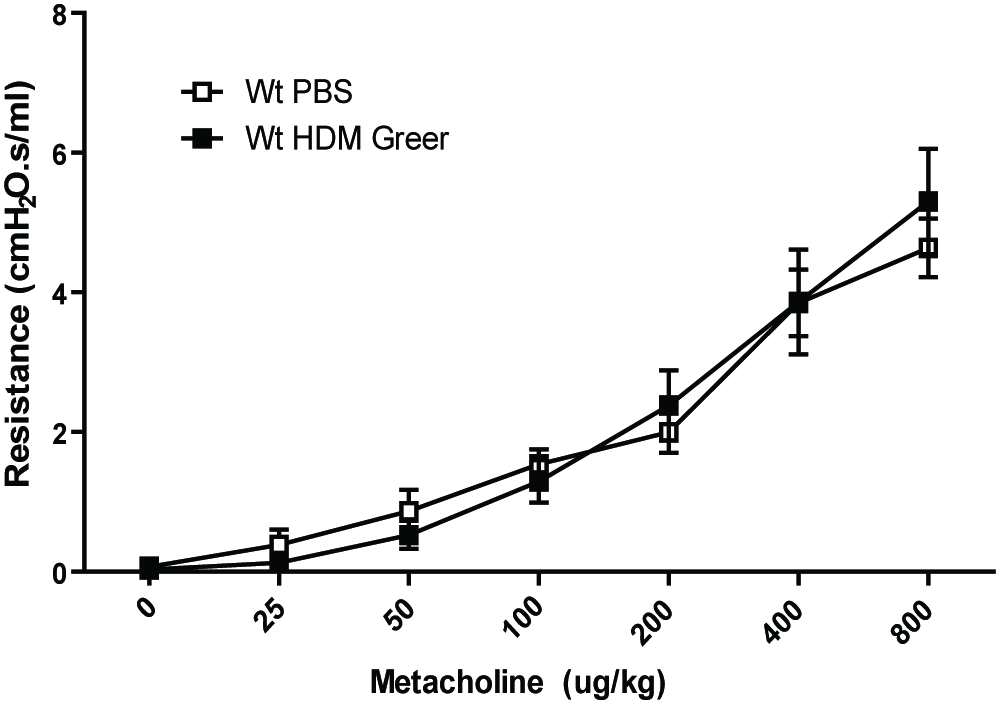

Supplement: Figure S1 — C57Bl/6J mice do not show significant differences in airway resistance after PBS or HDM exposure. Airway hyperreactivity was measured by Flexivent in response to metacholine in Wt C57Bl/6J mice after PBS and HDM Greer exposure. Absolute mean values (±SEM) are shown. (TIF) [file pone.0091206.s001.tif]

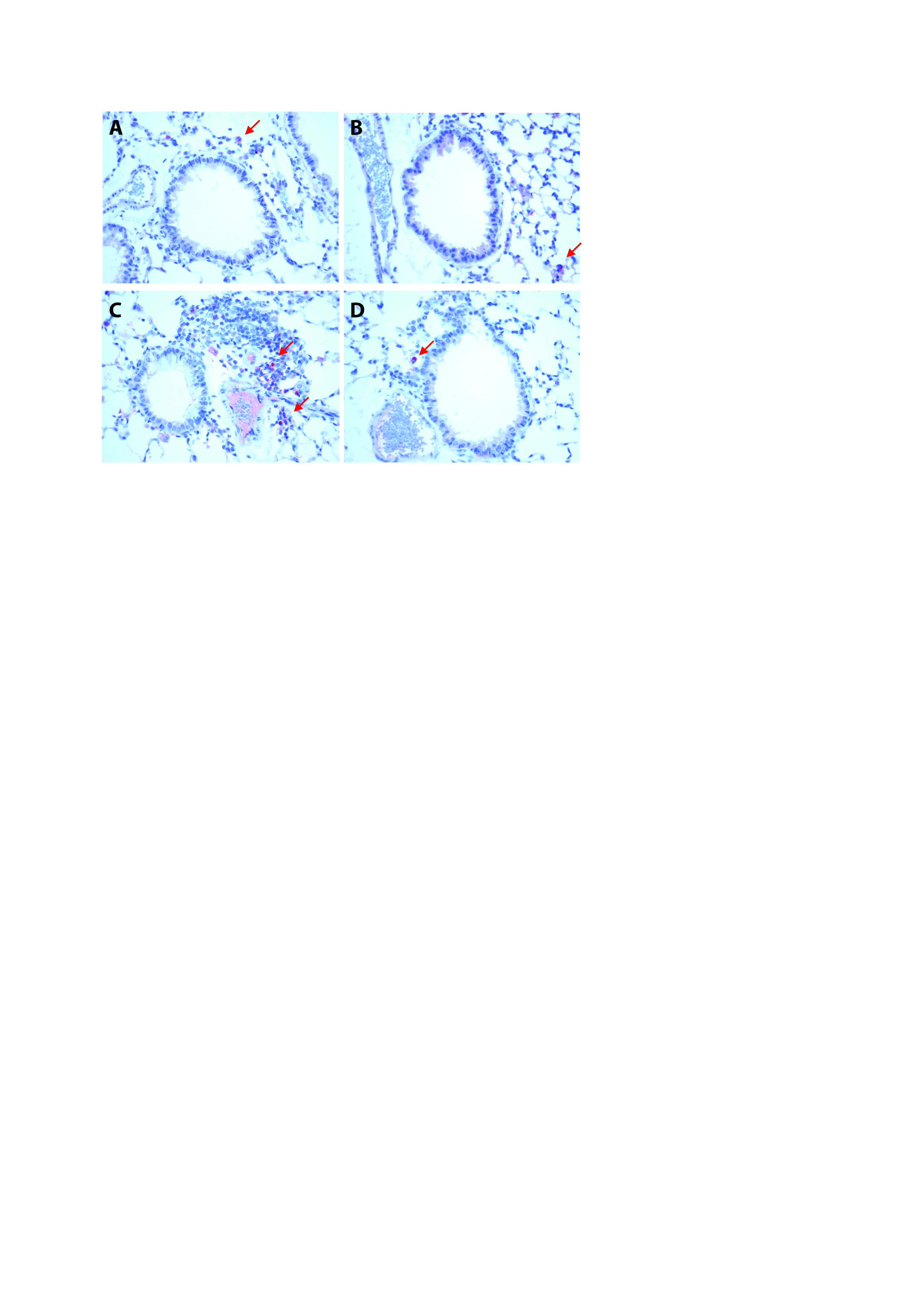

Supplement: Figure S2 — HDM exposure has no effect on Par-2 expression. PAR-2 staining of histological lung sections obtained from (A) BALB/c and (B) C57BL/6J mice exposed to PBS and BALB/c mice exposed to (C) Greer HDM extract and (D) Citeq HDM extract twice a week for a period of 5 weeks. Representative pictures are shown. Magnification 40×. Red arrows indicate highly PAR-2 expressing cells. (TIF) [file pone.0091206.s002.tif]
